# RISING STARS: Targeting G protein-coupled receptors to regulate energy homeostasis

**DOI:** 10.1530/JME-23-0014

**Published:** 2023-04-19

**Authors:** Aqfan Jamaluddin, Caroline M Gorvin

**Affiliations:** 1 Institute of Metabolism and Systems Research (IMSR) and Centre for Diabetes, Endocrinology and Metabolism (CEDAM), University of Birmingham, Birmingham, UK; 2 Centre of Membrane Proteins and Receptors (COMPARE), Universities of Birmingham and Nottingham, Birmingham, UK

**Keywords:** appetite, combinatorial therapeutics, diabetes, G protein, metabolism, obesity

## Abstract

G protein-coupled receptors (GPCRs) have a critical role in energy homeostasis, contributing to food intake, energy expenditure and glycaemic control. Dysregulation of energy expenditure can lead to metabolic syndrome (abdominal obesity, elevated plasma triglyceride, LDL cholesterol and glucose, and high blood pressure), which is associated with an increased risk of developing obesity, diabetes mellitus, non-alcoholic fatty liver disease and cardiovascular complications. As the prevalence of these chronic diseases continues to rise worldwide, there is an increased need to understand the molecular mechanisms by which energy expenditure is regulated to facilitate the development of effective therapeutic strategies to treat and prevent these conditions. In recent years, drugs targeting GPCRs have been the focus of efforts to improve treatments for type-2 diabetes and obesity, with GLP-1R agonists a particular success. In this review, we focus on nine GPCRs with roles in energy homeostasis that are current and emerging targets to treat obesity and diabetes. We discuss findings from pre-clinical models and clinical trials of drugs targeting these receptors and challenges that must be overcome before these drugs can be routinely used in clinics. We also describe new insights into how these receptors signal, including how accessory proteins, biased signalling, and complex spatial signalling could provide unique opportunities to develop more efficacious therapies with fewer side effects. Finally, we describe how combined therapies, in which multiple GPCRs are targeted, may improve clinical outcomes and reduce off-target effects.

## Invited Author’s profile


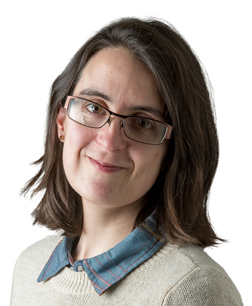


**Caroline Gorvin** is a Sir Henry Dale Fellow at the Institute of Metabolism and Systems Research (IMSR), University of Birmingham. She obtained her PhD in 2012 from the University of Oxford, where her research focussed on the cellular mechanisms by which mutations in a chloride-proton antiporter cause the renal disorder Dent’s disease. Caroline continued in Oxford undertaking postdoctoral research investigating the signalling and trafficking of the G protein-coupled receptor (GPCR), calcium-sensing receptor, and its role in calcium homeostasis. Caroline moved to the IMSR in 2018 to establish her research group investigating metabolic GPCRs. Her current research focusses on how metabolic GPCRs cross-talk and interact to regulate appetite and bone metabolism.

## Introduction

G protein-coupled receptors (GPCRs) are the largest family of transmembrane proteins and are involved in the regulation of diverse physiological processes including regulation of the cardiovascular, nervous and endocrine systems. GPCRs have a critical role in energy homeostasis, which can be understood as the balance between food intake and energy expenditure and is required for the proper functioning of fundamental cellular processes including cell division, growth and repair, protein synthesis and immune responses. Dysregulation of energy expenditure can lead to metabolic syndrome (abdominal obesity, elevated plasma triglyceride, LDL cholesterol and glucose, and high blood pressure), which is associated with an increased risk of developing obesity, diabetes mellitus, non-alcoholic fatty liver disease and cardiovascular complications. These chronic diseases are increasing in prevalence and understanding the molecular mechanisms by which energy expenditure is regulated is vital for the development of effective therapeutic strategies to treat and prevent these conditions.

**Figure 1:**
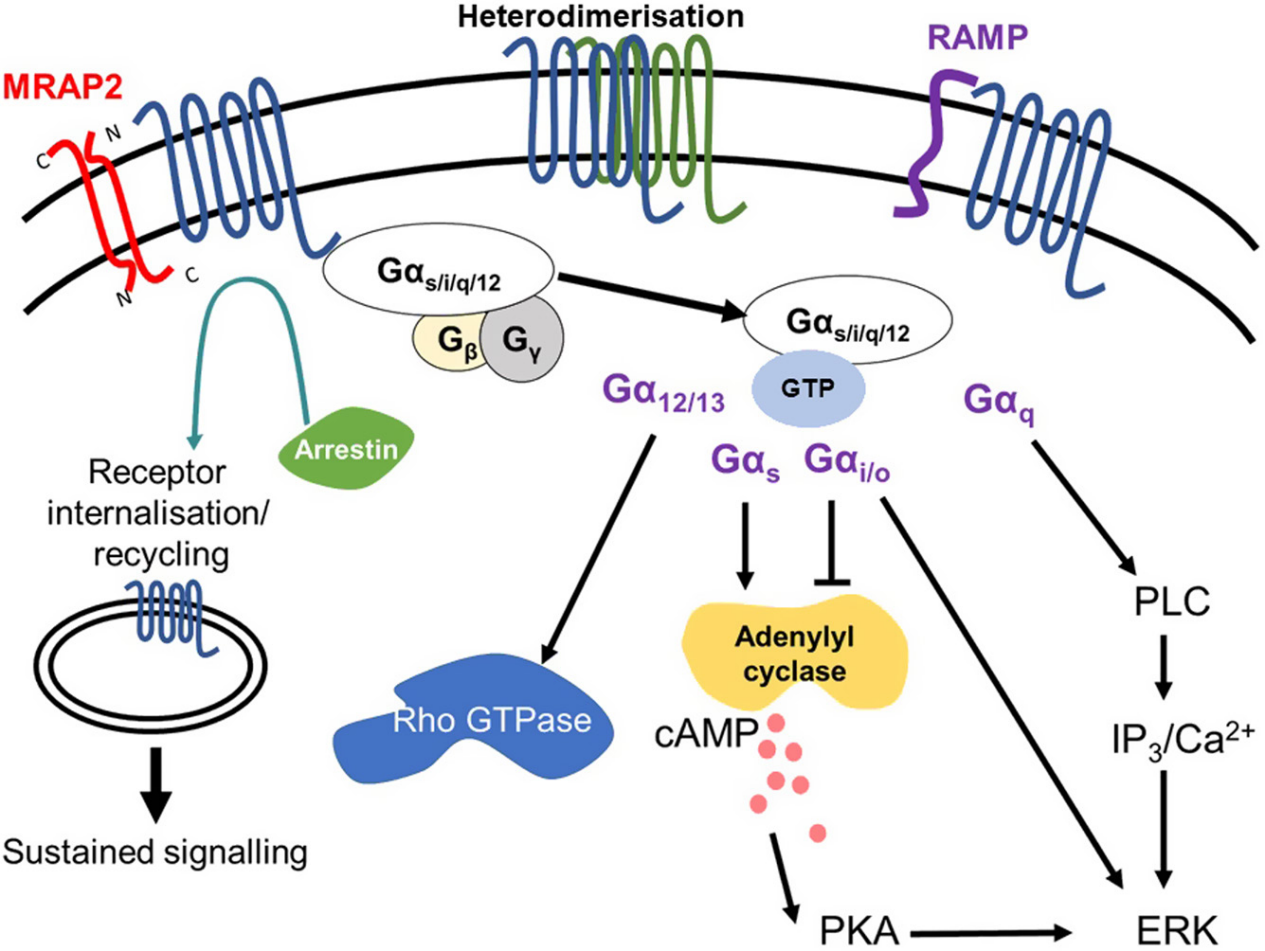
GPCR signalling pathways. At the cell surface, GPCRs can be modified by accessory proteins such as receptor activity-modifying protein (RAMP) and melanocortin 2 receptor accessory protein 2 (MRAP2) and may heterodimerise with other GPCRs. Agonist binding stabilises a GPCR conformation that interacts with the heterotrimeric G protein, comprising a Gα-subunit and a Gβγ subunit. Upon receptor activation, GDP–GTP exchange occurs at the Gα subunit, and Gα dissociates from Gβγ subunits. Both Gα (subfamily G_s_, G_i__/o_, G_q__/11_ and G_12/13_) and Gβγ then activate distinct signalling cascades. Only the Gα signalling pathways are shown. Gs activates the cAMP-dependent pathway, stimulating adenylyl cyclase to produce cAMP, which activates signalling partners such as protein kinase A (PKA). PKA phosphorylates several downstream targets including the extracellular signal-regulated kinase (ERK) signalling pathway. The G_i__/o_ family primarily inhibits adenylyl cyclase. G_12/13_ regulates Rho GTPase activity. The G_q__/11_ family activates phospholipase C (PLC), which cleaves phosphatidylinositol 4,5-bisphosphate (PIP2) into inositol 1,4,5-trisphosphate (IP_3_) and diacylglycerol (DAG). IP_3_ stimulates increases in cytosolic calcium (Ca^2+^) and DAG stimulates ERK signalling. Recruitment of β-arrestin facilitates internalisation and can initiate signalling for some receptors. Following internalisation, GPCRs enter the endosomal–lysosomal pathway or are targeted to the recycling pathway. Some GPCRs are also able to signal from internal compartments including endosomes and the trans-Golgi network (sustained signalling).

GPCRs are effective pharmacological targets as they are cell surface expressed and account for ~30% of the global market share of therapeutic drugs. In recent years, drugs targeting GPCRs have been the focus of efforts to improve treatments for type 2 diabetes and obesity, and increasingly targeting multiple GPCRs with physiological and/or pharmacological overlap has become the focus of efforts by the pharmaceutical industry. Moreover, as we have learned about the complexity of GPCR signalling, including modulation upon binding to interacting proteins, biased signalling and signalling from intracellular compartments (Fig. 1), new pathways have emerged that could provide unique opportunities to develop more efficacious therapies with fewer side effects.

**Figure 2:**
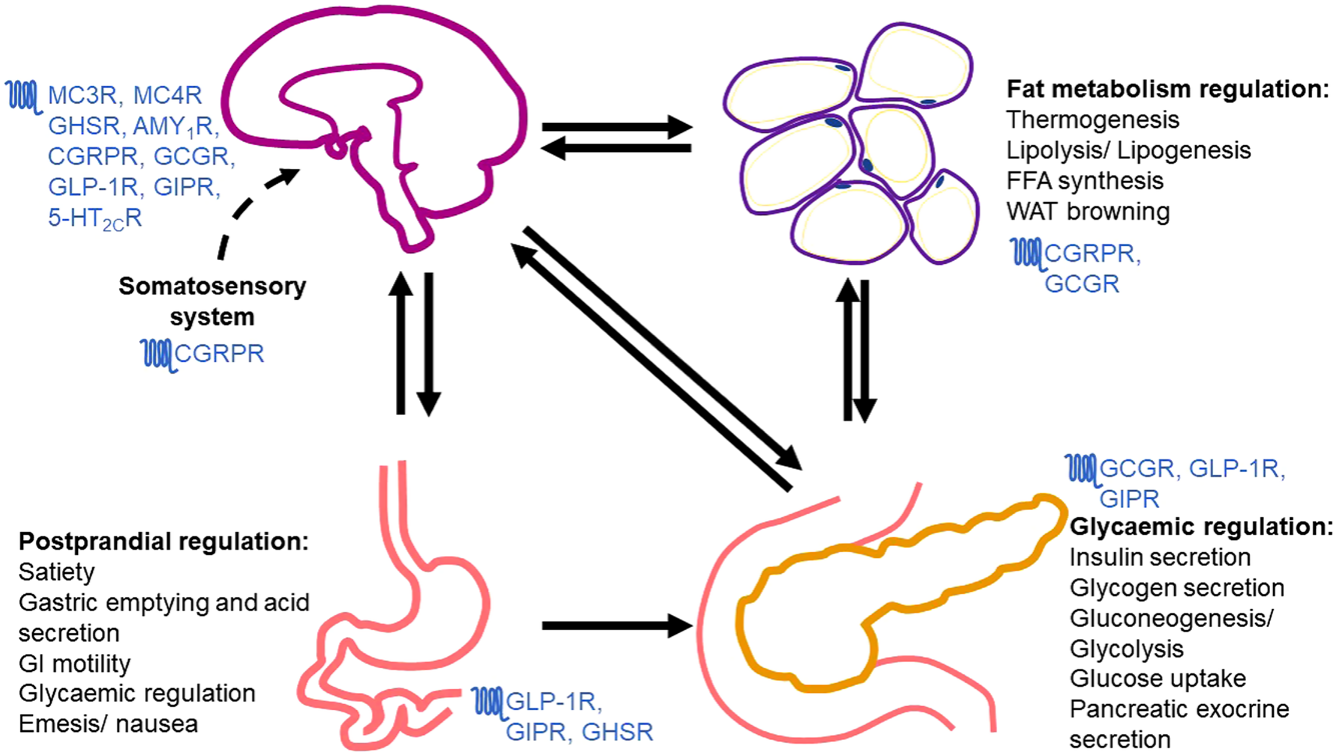
Primary tissues and organs involved in energy homeostasis. Postprandial signals from the gut and vagal afferent neurons innervating the stomach, intestines, liver and pancreas relay signals to the brainstem to regulate whole-body functions. Changes in blood glucose levels are monitored by the pancreatic islet cells which function in a feedback loop regulated primarily by the insulin and glucagon hormones. Insulin and glucagon enter the brain from the circulation to activate hypothalamic neurons, which induces either anorexigenic or orexigenic effects. Peripheral adipose tissue thermogenesis and fat metabolism regulation are dependent on sympathetic outflow to it but can also signal back to the CNS or other organs through circulating leptin and adiponectin derived from adipose tissues to improve glycaemic regulation and overall energy homeostasis. The somatosensory system, primarily associated with temperature, haptic and pain sensations, may also provide information to the brain that inadvertently induces a response in energy homeostasis for example, cold-induced thermogenesis.

In this review, we focus on nine GPCRs with roles in energy homeostasis (Table 1, Fig. 2) that are current and emerging targets to treat obesity and diabetes and describe how new insights into how these receptors signal could provide unique pharmacological targets for future therapies.

**Table 1:** Overview of major GPCRs involved in energy homeostasis.

Receptor	Major sites of expression/relevant activity	Endogenous ligand/s	Major signalling pathway	Physiological functions	Approved drugs
**Melanocortin axis receptors**					
Melanocortin receptor 4 (MC4R)	Hypothalamus – PVN (Yeo *et al.* 2021)	αMSH, βMSH, γMSH (agonists) AgRP (antagonist)	Gs (major), Gq/11 Modified by MRAP2	Satiety	Setmelanotide for obesity Bremelanotide for use in women with low sexual desire
Melanocortin receptor 3 (MC3R)	Hypothalamus – ARC (AgRP/POMC neurons) (Ghamari-Langroudi *et al.* 2018, Sweeney *et al.* 2021)		Gs	Bidirectional responses to calorie restriction and calorie intake, growth, onset of puberty	None^a^
Growth hormone secretagogue receptor 1a (GHSR1a)	Hypothalamus – ARC (NPY/AgRP neurons) (Müller *et al.* 2015), Vagal afferent neurons innervating GI tract (Davis *et al.* 2020), Pancreatic cells (Yin *et al.* 2020)	Ghrelin (agonist) LEAP2 (antagonist)	Gq Modified by MRAP2	Growth hormone secretion, appetite stimulation, insulin release, adiposity, gastric emptying	None^a^
**Calcitonin family receptors**					
Amylin receptor (AMY_1/2/3_R)	Hypothalamus, Brainstem (Zakariassen *et al.* 2020)	Amylin, CGRP	Gs/Gq	Glycaemic regulation, satiety, gastric emptying	Pramlintide for use in type 1 & type 2 diabetes
Calcitonin gene-related peptide (CGRPR)	Adipose tissue (Makwana *et al.* 2021), Midbrain/pons parabrachial nucleus-amygdala/BNST, Peripheral somatosensory neurons (Campos *et al.* 2016)	CGRP	Gs/Gq	Satiety, vasodilation, lipid homeostasis, glycaemic regulation	Monoclonal antibodies and antagonists approved for migraine
**Glucagon family receptors**					
Glucagon receptor (GCGR)	Liver (Geary *et al.* 1993), Kidney (Ouhilal *et al.* 2012), Hypothalamus – ARC (Quiñones *et al.* 2015)	Glucagon	Gs, Gq	Glycaemic regulation, may have some roles in satiety, lipid homeostasis, thermogenesis	None^a^
Glucagon-like peptide-1 receptor (GLP-1R)	Pancreatic β-cells (Portha *et al.* 2011, McLean *et al.* 2021), Vagal neurons innervating GI tract (Williams *et al.* 2016), Hypothalamus – ARC (He *et al.* 2019)	GLP-1	Gs	Glycaemic regulation, satiety	GLP-1R only: Exenatide, Liraglutide, Lixisenatide, Dulaglutide, Semaglutide, Combined GLP-1R & GIPR: Tirzepatide
Glucose-dependent insulinotropic polypeptide receptor (GIPR)	Pancreatic α- & β-cells (Widenmaier *et al.* 2010, McLean *et al.* 2021) Vagal neurons innervating GI tract (Kim *et al.* 2012), Hypothalamus – ARC, PVN, DMH (Zhang *et al.* 2021)	GIP	Gs	Glycaemic regulation, satiety	Combined GLP-1R & GIPR: Tirzepatide
**5-HT family receptors**					
5-hydroxytryptamine (5-HT, serotonin) receptor 2C (5HT2CR)	Hypothalamus – ARC, NTS (D'Agostino *et al.* 2018)	5-HT	Gq	Satiety	Lorcaserin (withdrawn) D-fenfluramine (withdrawn) Sibutramine (withdrawn)

^a^Several therapeutic drug candidates have been identified or entered clinical trials but have yet to be approved for therapeutic use.

ARC, arcuate nucleus; BNTS, bed nucleus of the stria terminalis; DMH, dorsomedial hypothalamus; NTS, nucleus of the solitary tract; PVN, paraventricular nucleus.

## Melanocortin axis receptors

### Melanocortin receptors

**Figure 3:**
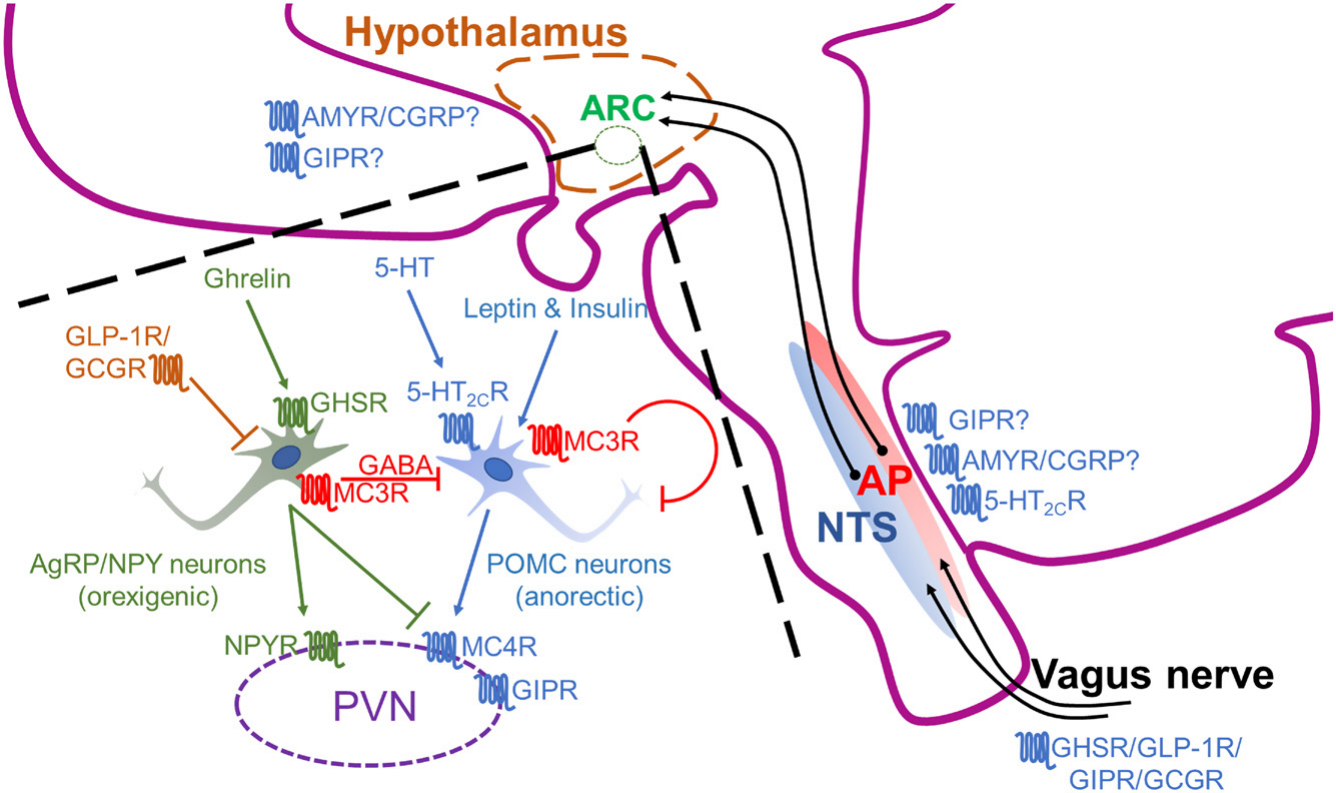
GPCR expression and function in the hypothalamus, with associated neuronal pathways linked to the hypothalamus. Hormonal peptides are secreted into the circulation and can act locally on and propagate signals towards the CNS via the vagus nerve. The solitary tract nucleus (NTS) receives and integrates afferent vagal information and innervates a wide variety of brain regions including the hypothalamus. Some circulating hormones such as insulin, glucagon, leptin and amylin can also cross the blood–brain barrier and bind to their respective receptors in the CNS, primarily in the hypothalamus. The arcuate nucleus (ARC) regulates satiety, energy expenditure and glycaemic control. Two major populations within the ARC respond to nutrient input to regulate appetite: the anorexigenic pro-opiomelanocortin-expressing neurons (POMC) that release αMSH and orexigenic agouti-related peptide and neuropeptide Y (AgRP/NPY) neurons that release AgRP and NPY. AgRP/NPY neurons also inhibit POMC neurons via GABA. Signals converge at the paraventricular nucleus (PVN) where αMSH stimulates MC4R to reduce appetite, while NPY activates NPY receptors (NPYR) and AgRP inhibits MC4R to induce appetite. There are multiple GPCR expressions at various regions of the neuronal circuit that serve to modulate both the orexigenic and anorexigenic stimulus. A question mark has been inserted where a role has been proposed, but there is still some need for verification.

The melanocortin system communicates hormonal signals from peripheral tissues such as the pancreas and adipose tissue to the central nervous system and is responsible for regulating adrenal development and sexual behaviour, as well as energy homeostasis. The melanocortin peptide hormones (α, β and γ melanocyte-stimulating hormone (MSH) and adrenocorticotrophin hormone) are produced as post-translational products from the prohormone pro-opiomelanocortin (POMC) and act upon five melanocortin receptors (MC1R–MC5R), while two endogenous antagonists, agouti and agouti-related peptide (AgRP), suppress the receptors actions (Yeo *et al.* 2021). The MC3R and MC4R are the most important in energy expenditure. POMC is released from neurons of the hypothalamic arcuate nucleus (ARC) in response to hormonal signals such as leptin. The biologically active products αMSH and βMSH act on MC4R on the paraventricular nucleus (PVN) to increase cAMP signals and suppress food intake (Fig. 3). AgRP and neuropeptide Y (NPY)-expressing ARC neurons promote food intake by inhibiting MC4R and activating NPY-receptors on the PVN, respectively (Yeo *et al.* 2021). NPY/AgRP neurons can also antagonise POMC neurons through γ-aminobutyric acid (GABA) signalling. Insulin and leptin suppress AgRP/NPY neurons, while ghrelin stimulates these neurons.

Human genetic studies have confirmed the importance of MC4R in the central regulation of energy homeostasis. Heterozygous loss-of-function mutations in MC4R are the most common monogenic form of obesity. Most inactivating MC4R mutations are missense and have poor cell-surface expression due to intracellular retention at the endoplasmic reticulum (Granell *et al.* 2010). Until recently, up to 25% of MC4R variants identified in obese cohorts were classified as non-pathogenic as they did not affect cAMP production. However, comprehensive *in vitro* investigation has since shown that these variants impair MC4R endocytosis, receptor dimerisation and/or G protein coupling (Brouwers *et al.* 2021). This indicates that targeting receptor dimerisation using bitopic ligands, or promoting G-protein coupling using allosteric modulators, could be effective treatments for early-onset obesity. Moreover, *in vitro* investigations have shown that many mutant MC4Rs that are ER-retained do not impair receptor functionality, indicating that increased trafficking of these receptors to the cell surface by pharmacochaperones could be a viable treatment for hereditary obesity (Granell *et al.* 2010).

A subset of MC4R variants, identified in the UK Biobank, caused a gain in receptor function, and are associated with significantly lower BMI and lower odds of obesity, type-2 diabetes and coronary artery disease (Lotta *et al.* 2019). These variants increased receptor activity by biasing signalling towards β-arrestin recruitment and increased mitogen-activated protein kinase pathway signalling, indicating that drugs that preferentially activate these MC4R pathways may be effective in reducing weight and treating obesity-related disease (Lotta *et al.* 2019).

Melanocortin receptor signalling can be modulated by interactions with accessory proteins such as melanocortin-2 receptor accessory protein 2 (MRAP2), a heteromeric single-pass transmembrane protein, which enhances MC4R signalling. Global and *Mc4r* neuron-specific inactivation of MRAP2 increased food intake, weight gain and adiposity, and inactivating human* MRAP2* variants have been identified in patients with hyperphagic obesity with hyperglycaemia and hypertension (Baron *et al.* 2019). Targeting MRAP2 could be effective in improving MC4R signalling, although the effect on other MRAP2-interacting GPCRs, including prokineticin and ghrelin receptors (Srisai *et al.* 2017) must be considered. Further studies to decipher how MRAP2 regulates different GPCRs, and how these interactions affect food intake and adiposity will help inform drug development strategies for MRAP2.

Efforts to target the melanocortin system have been hampered by adverse effects caused by a lack of melanocortin receptor selectivity or pathway specificity. Tachycardia and hypertension are of particular concern, as fusion of a highly-selective MC4R agonist led to increases in blood pressure and heart rate (Greenfield *et al.* 2009). However, studies of mice in which specific G proteins were deleted from the PVN showed that distinct pathways regulate MC4R-mediated effects on food intake and blood pressure. Thus, the ability of an MC4R agonist to inhibit food intake was lost in mice with PVN-deletion of Gq/11, while the agonist’s effect on blood pressure was lost in animals lacking Gs at the PVN (Li *et al.* 2016). As such, ligands that bias MC4R signalling to favour Gq/11 should suppress appetite while avoiding cardiovascular effects. Setmelanotide is the first approved anti-obesity drug for the treatment of rare genetic conditions associated with obesity and binds to MC4R with high affinity. Setmelanotide decreased food intake and adiposity, improved glucose tolerance and was associated with persistent weight loss in non-human primates, without effect on blood pressure and heart rate (Kievit *et al.* 2013). Moreover, setmelanotide treatment also increased weight loss in obese people with MC4R mutations (Collet *et al.* 2017). *In vitro* studies have indicated that setmelanotide is more efficacious in Gq/11-PLC activation than Gs-activation, which may explain why the drug is not associated with adverse effects on blood pressure observed with other MC4R agonists (Heyder *et al.* 2021).

Another MC4R agonist, bremelanotide, which also binds to MC3R, has recently been approved in the United States for use in premenopausal women with low sexual desire. Data from two small randomised-control studies of obese premenopausal women showed that bremelanotide reduced caloric intake and weight loss, while having no adverse effects on blood pressure (Spana *et al.* 2022), indicating this may be another effective treatment for obesity. Antagonism of MC4R has also been investigated to treat the metabolic and dietary components of cachexia and anorexia that lead to tissue wasting. The peptide antagonist, TCMCB07, showed beneficial effects of increased food intake, body weight and preserved fat mass and lean mass in three rat models of cachexia and anorexia (Zhu *et al.* 2020). Moreover, another small molecule, PF-07258669, showed potent MC4R antagonism in an aged model of cachexia (Garnsey *et al.* 2023) and is currently being investigated in clinical trials.

Elucidating the physiological functions of MC3R has proven more difficult than that of MC4R. While early studies of MC3R in rodent models indicated roles for MC3R in energy metabolism, including in food anticipatory responses, and associations between MC3R deletion and mild late-onset obesity without hyperphagia or hypometabolism (Sutton *et al.* 2008), other studies produced seemingly contradictory findings. Studies in the past decade have shown that MC3R is required bidirectionally to control responses to both calorie restriction and calorie intake (Ghamari-Langroudi *et al.* 2018, Sweeney *et al.* 2021). Despite expression on both POMC and AgRP neurons, MC3R feeding effects are mediated predominantly by AgRP expression, supporting a model in which MC3R agonists increase food intake by stimulating GABA release from AgRP neurons, to suppress MC4R activity at the PVN (Ghamari-Langroudi *et al.* 2018, Sweeney *et al.* 2021).

Following the identification of inactivating MC4R mutations in obesity, several studies investigated genetic variants in MC3R in obese individuals, with inconsistent findings. A recent study investigating rare MC3R variants identified in the UK Biobank identified several loss-of-function MC3R variants that were associated with delayed puberty and reduced linear growth and lean mass (Lam *et al.* 2021). The authors suggested that MC3R may provide a link between nutritional status and linear growth and the onset of obesity.

Small molecules that act specifically on the MC3R have recently been described, with MC3R agonists shown to increase feeding and reduce anxiety, and MC3R-specific antagonists shown to suppress feeding in mice (Sweeney *et al.* 2021). This is consistent with studies showing MC3R-depletion enhances anorexia in mice (Sweeney *et al.* 2021) and indicates that modulating MC3R could be an effective therapy to increase weight gain and reduce anxiety in eating disorders while antagonising the receptor could promote weight loss. Additionally, MC3R agonists could be effective in patients with delayed puberty and/or short stature (Lam *et al.* 2021, Sweeney *et al.* 2021). Studies in humans examining the effects of these MC3R agonists and antagonists will be required to assess their effect on multiple parameters including weight, food intake, anxiety, growth, onset of puberty and cardiovascular functions (e.g. blood pressure and heart rate).

### Ghrelin receptor

The growth hormone secretagogue receptor (GHSR1a) is a Gq-coupled receptor for the gut-derived hormone ghrelin, which has multiple roles in energy homeostasis including growth hormone secretion, stimulation of appetite, energy expenditure, insulin release, adiposity and gastric emptying (Müller *et al.* 2015). Within the hypothalamus, GHSR1a is expressed predominantly in NPY/AgRP neurons and ghrelin mediates its orexigenic effect by increasing NPY and AgRP expression and hyperpolarising POMC neurons, most likely mediated by the GABAergic NPY/AgRP neurons (Müller *et al.* 2015). Ghrelin and leptin have a reciprocal relationship aimed at increasing or decreasing adiposity. Fasting increases ghrelin while reducing leptin, and high leptin levels suppress gastric ghrelin secretion and prevent ghrelin-induced NPY neuron action (Kalra *et al.* 2005). GHSR1a is also expressed in several non-hypothalamic brain areas where it may function in taste sensation, reward behaviours and olfaction, all of which could contribute to ghrelin’s effect on food intake (Müller *et al.* 2015).

As ghrelin secretion is increased during fasting and diminishes post-prandially in mice and humans, it was hypothesised that ghrelin may have a role in food anticipation and that blocking its actions may reduce food intake, adiposity and blood glucose (Mani *et al.* 2019). However, deletion of the GHSR or ghrelin gene from mice had little effect on body weight or insulin and leptin levels, although some differences were observed when mice were placed on a high-fat diet, likely due to ghrelin’s role in fat utilisation (Müller *et al.* 2015). Moreover, ghrelin levels are low in obesity and binge eaters, while the orexigenic effect of ghrelin is less potent in obese mice, which indicated that a counter-regulatory mechanism may exist to limit ghrelin activity when food and nutrients are available (Müller *et al.* 2015, Mani *et al.* 2019). In 2018, an endogenous peptide, liver-expressed antimicrobial peptide 2 (LEAP2), which acts as an inverse agonist to GHSR1a constitutive activity and a competitive ghrelin antagonist, was discovered and appears to fulfil this counter-regulatory mechanism. LEAP2 is produced in the liver and small intestine and impairs ghrelin-induced food intake, growth hormone release and increases in blood glucose (Ge *et al.* 2018). LEAP2 is suppressed by fasting and is found at high levels in diet-induced obese mice and obese individuals, indicating that the plasma LEAP2/ghrelin ratio may be a key determinant in responses to feeding status, body mass and glucose (Mani *et al.* 2019). The discovery of LEAP2 has reinvigorated interest in targeting the GHSR1a to control appetite. Prior to this, GHSR1a antagonists, inverse agonists and antibodies designed to neutralise ghrelin had little long-term success in animal studies and clinical trials, with food intake quickly resuming in those studies in which an initial suppression of appetite was reported. However, a recent small clinical trial in 20 healthy individuals showed that intravenous administration of LEAP2 could successfully reduce food intake, meal duration and postprandial glucose, presenting the possibility that LEAP2 could be of clinical utility in type-2 diabetes and obesity (Hagemann *et al.* 2022). Further trials will be required to investigate the long-term effects of LEAP2 on food intake and glucose concentrations, and effects in patients with diabetes and obesity need to be investigated.

GHSR1a activity can also be modified by binding to accessory proteins such as MRAP2. Mice with targeted deletion of *Mrap2* from AgRP neurons lose their ghrelin-induced increase in food intake (Srisai *et al.* 2017). *In vitro* studies showed that MRAP2 elicits signalling bias by enhancing Gq/11-mediated signalling, while inhibiting β-arrestin-mediated RhoA signalling and GHSR1a constitutive activity (Rouault *et al.* 2017, Srisai *et al.* 2017). This indicates that drugs that disrupt the GHSR1a and MRAP2 interaction sites may be more effective in reducing food intake than those targeting GHSR1a or MRAP2 alone. MRAP2 also regulates ghrelin-mediated reductions in insulin secretion by directly binding to GHSR1a in pancreatic δ-cells to facilitate ghrelin-induced somatostatin release that inhibits insulin release from pancreatic β-cells (Yin *et al.* 2020). Such ghrelin-mediated effects on insulin are protective mechanisms to prevent hypoglycaemia during fasting. Inhibition of GHSR1a with a selective antagonist has been shown to prevent ghrelin-mediated suppression of insulin release and improve glucose tolerance in obese mice (Esler *et al.* 2007). Therefore, GHSR1a antagonists or compounds that disrupt the GHSR1a–MRAP2 interaction site could be effective in promoting glucose-dependent insulin secretion in type-2 diabetics.

Finally, GHSR1a has been described to form heteromers with several GPCRs, which modify GHSR1a signalling. Although most interactions have been identified in cell lines and their physiological significance is unknown, interactions between GHSR1a and dopamine receptor-1 (DRD1) have been explored in detail in relevant tissues and animal models. GHSR1a enhances DRD1-induced initiation of hippocampal synaptic plasticity and formation of hippocampal memory, and these complexes are disrupted in Alzheimer’s disease (Tian *et al.* 2019). Targeting GHSR1a-DRD1 heteromers with a bitopic agonist could be an important preventative treatment for Alzheimer’s disease, although the effects on food intake and insulin secretion would need to be assessed carefully.

### Calcitonin family receptors

The calcitonin family of peptides comprises calcitonin, amylin, α- and β-calcitonin gene-related peptide (CGRP) and adrenomedullin 1 and 2, which regulate diverse physiological functions including bone metabolism, appetite, nutrient utilisation and vasodilation (Hay *et al.* 2018). While calcitonin binds to the calcitonin receptor (CTR), the other peptides bind to distinct receptors consisting of a class B GPCR (CTR or CTR-like receptor (CLR)) heterodimerised with a receptor activity-modifying protein (RAMP), which is required for receptor trafficking to cell surfaces (Hay *et al.* 2018). Combination of the CTR with RAMP1, 2 or 3 produces AMY_1_, AMY_2_ and AMY_3_ receptors, respectively, which bind amylin, while CLR combined with RAMP1 forms the CGRP receptor, and CLR with RAMP2 and RAMP3, the receptors for adrenomedullin 1 and 2, respectively (Hay *et al.* 2018). Amylin and CGRP receptors are most relevant to energy metabolism.

### Amylin receptor

Amylin is a pancreatic hormone that is synthesised and released alongside insulin from pancreatic β-cells and has roles in satiety, gastric emptying and glucagon secretion. Due to a paucity of highly selective antagonists to individual receptors and the ability of CGRP to bind to amylin receptors, it remains difficult to differentiate the effects of individual amylin receptors *in vivo* (Hay *et al.* 2018). A recent study would suggest that AMY_1_ receptor may be responsible for fat utilisation and storage, while AMY_3_ receptor is responsible for glycaemic regulation, although this study used RAMP knockout animal models, which would also eliminate CGRP receptor expression (Coester *et al.* 2020).

Amylin is released post-prandially and acts as a satiation signal binding to amylin receptors predominantly expressed in the area postrema and nucleus of the solitary tract (NTS) in the brainstem (Zakariassen *et al.* 2020). Amylin activity in the ventral tegmental area in the midbrain and nucleus accumbens in the forebrain may be linked to reducing rewarding effects from food. Amylin and leptin act synergistically, which may, in part, be due to amylin acting directly on ARC NPY/AgRP neurons co-expressing leptin receptor (Zakariassen *et al.* 2020). Amylin’s ability to slow post-prandial gastric emptying also contributes to satiety. Amylin also reduces the rate of glucose absorption and inhibits glucagon secretion (Gedulin *et al.* 2006).

Amylin’s beneficial effects on blood glucose and weight loss led to the development of a stable amylin analogue, pramlintide, approved for use in diabetes mellitus as an adjunct to insulin. When administered to people with obesity, pramlintide improved weight loss, while in patients with type-1 diabetes mellitus, it improved hyperglycaemia and delayed gastric emptying (Aronne *et al.* 2010). Pramlintide is generally well tolerated, although gastrointestinal side effects can occur, and its short half-life necessitates multiple daily injections. This led to the development of the long-acting amylin analogue cagrilintide. A recent clinical trial reported that once-weekly injection of cagrilintide significantly reduced body weight in people with a BMI >30 kg/m^2^ (Lau *et al.* 2021).

### CGRP receptor

CGRP is a product of alternative splicing of the calcitonin gene, expressed in the central and peripheral nervous systems and is a potent vasodilator. It is increased in headache disorders and its receptor is the target of monoclonal antibodies and antagonists for the treatment of migraine. Shortly after its discovery, CGRP was also reported to affect energy homeostasis with elevated levels reported in obese humans and animals (Liu *et al.* 2017*b*). However, deciphering CGRP function in food intake and appetite has proven difficult with animal models producing contradictory findings. Intraperitoneal administration of CGRP to mice significantly decreased food consumption and lowered energy expenditure (Sanford *et al.* 2019). However, CGRP knockout mice fed a high-fat diet have significantly lower body weight, visceral fat and serum leptin, suggesting these animals are protected from diet-induced obesity (Liu *et al.* 2017*b*). Additionally, genetic ablation of heat-sensing CGRPα neurons promoted resistance to weight gain and increased energy expenditure by enhanced expression of fatty acid oxidation genes, higher *ex vivo* lipolysis in primary white adipocytes, and increased mitochondrial respiration from interscapular brown adipose tissue (iBAT) (Makwana *et al.* 2021). However, other animal models identified no changes upon CGRP deletion.

CGRP has been implicated in the control of meal termination, regulated by CGRP neurons that are directly innervated by AgRP-neurons (Campos *et al.* 2016). As CGRP regulates meal termination while other receptors regulate food intake (e.g. MC4R), it is feasible that targeting both pathways would have greater benefits than one pathway alone. However, a greater understanding of how CGRP signalling regulates vasodilation vs food intake will be required to avoid off-target effects (e.g. headaches) of potential CGRP agonists.

CGRP may also regulate glycaemic control. CGRP reduced glucose-stimulated insulin secretion and increased glucagon in animals (Nilsson *et al.* 2016), while deletion of the CGRP receptor in mice improved glucose tolerance and insulin sensitivity (Liu *et al.* 2017*b*). However, other studies were unable to confirm these effects on insulin (Sanford *et al.* 2019). Differences in findings could be explained by some studies using supraphysiological concentrations of CGRP, or due to the effects of amylin, which can also bind to CGRP receptors. More promisingly, a monoclonal antibody against CGRP was recently shown to lower hyperglycaemia and adiposity in a type-2 diabetes mouse model, suggesting targeting CGRP could have therapeutic benefits in diabetes (Halloran *et al.* 2020).

## Glucagon family receptors

### Glucagon receptor

The glucagon receptor (GCGR) binds the hormone glucagon, secreted by pancreatic α-cells, which increases glucose and fatty acid concentrations. GCGR activity stimulates glucose production by increasing hepatic gluconeogenesis and glycogenolysis (Janah *et al.* 2019). Due to these actions on insulin and glucose, suppression of glucagon action was initially considered a good strategy to treat diabetes. However, animal studies have shown that hypothalamic glucagon signalling inhibits hepatic glucose production and suggested that hypothalamic glucagon resistance disrupts glucose homeostasis in diabetes and obesity (Mighiu *et al.* 2013). Moreover, glucagon stimulates energy expenditure, reduces food intake and enhances weight loss in rodent and human studies, which suggests glucagon agonism would be beneficial in obesity.

The mechanism by which glucagon and GCGR influence weight loss is incompletely understood. There is some evidence that in BAT, GCGR promotes thermogenesis through stimulation of lipid oxidation and free fatty acid synthesis, expression of thermogenic genes, increased body core temperature and oxygen consumption (Townsend *et al.* 2019), while GCGR expressed in white adipose tissue (WAT) can also lead to the ‘browning’ of WAT by expression of thermogenic genes (Townsend *et al.* 2019). However, a recent mouse model in which *Gcgr* was deleted from WAT showed no effects on body weight or glucose homeostasis and that physiological levels of glucagon did not regulate lipolysis (Vasileva *et al.* 2022). Moreover, a recent study using mice with deletion of *Gcgr* from BAT showed the receptor was not required for control of body weight, adiposity, core temperature or energy expenditure (Beaudry *et al.* 2019). Liver-derived FGF21, which is increased by acute and chronic GCGR activation may contribute to glucagon-mediated weight loss. Global deletion of FGF21 or neuronal deletion of its obligate co-receptor β-klotho from mouse partially impaired the effect of a GCGR agonist on weight loss demonstrating a central regulation of GCGR-mediated weight loss (Nason *et al.* 2021). It is likely that other mechanisms also contribute to glucagon-mediated weight loss.

Glucagon receptor activity also induces satiety and reduces meal size (Geary *et al.* 1993). In the liver, GCGR activity results in a satiety signal via the vagal afferent nerves to the NTS neuronal region in the brainstem, and subsequently a neuronal signal to the hypothalamus (Fig. 3) (Geary *et al.* 1993). Glucagon can also diffuse through the blood–brain barrier and bind directly to the GCGR expressed in the hypothalamic ARC (Quiñones *et al.* 2015). Hypothalamic GCGR activity inhibits AgRP-expressing neuron activity, thereby attenuating orexigenic effects, whereas central resistance to glucagon-induced hypophagia contributes to the development of obesity (Quiñones *et al.* 2015). However, further work is needed to properly differentiate the pharmacology of central GCGR and the closely-related glucagon-like peptide-1 receptor.

### GLP-1 receptor

Glucagon-like peptide-1 (GLP-1) and glucose-dependent insulinotropic polypeptide (also known as gastric inhibitory peptide, GIP) are gut-derived hormones known as incretins, as they promote the secretion of insulin. GLP-1 is processed from the pro-glucagon gene which contains sequences for glucagon, GLP-1 and GLP-2. The receptors for GLP-1 and GIP (GLP-1R and GIPR, respectively) are class B GPCRs with large extracellular domains (ECD), and the peptides bind in a two-step mechanism to both the ECD and the transmembrane domain. GLP-1R and GIPR are highly expressed in pancreatic β-cells where they promote glucose-stimulated insulin secretion (McLean *et al.* 2021). GLP-1 also slows gastric emptying and increases satiety.

GLP-1R activates cAMP pathways including PKA and EPAC2 to stimulate insulin secretory granule exocytosis from pancreatic β-cells. GLP-1R activation promotes proliferation and suppresses apoptosis of β-cells, thereby preserving β-cell mass, which could further improve glycaemic control (Portha *et al.* 2011). GLP-1 can suppress glucagon secretion, although the mechanism and whether GLP-1R is involved remain unresolved (McLean *et al.* 2021).

The desirable effects of GLP-1 on glycaemic control led to the development of a series of GLP-1 mimetics with a longer half-life than natural GLP-1 that bind and activate GLP-1R for use in the treatment of diabetes. A series of GLP-1R agonists with resistance to dipeptidyl dipeptidase-IV breakdown are used routinely to treat type-2 diabetes (Table 1) and can be administered daily to weekly (Khoo & Tan 2020). Liraglutide and semaglutide were subsequently approved for managing weight loss in obesity when appreciable losses of weight were observed in patients with type-2 diabetes (Khoo & Tan 2020). GLP-1R agonists have also been shown to improve cardiovascular complications such as stroke, myocardial infarction and cardiovascular death and are approved for use in type-2 diabetics at high risk of cardiovascular disease (Khoo & Tan 2020). Beyond metabolic disease, GLP-1R agonists have been investigated in a range of tissues and diseases including, Alzheimer’s disease, Parkinson’s disease, adrenal function, opioid dependency, bone turnover, angiogenesis and chronic pain. A detailed review of these studies is beyond the scope of this review and interested readers are referred to a recent publication detailing many of these (Zhao *et al.* 2021).

GLP-1R agonists are associated with gastrointestinal side effects at high doses, which limits their effectiveness in weight loss and reduces patient compliance. Therefore, improved therapies will be required to target GLP-1R while reducing these off-target effects. Current strategies include designing novel GLP-1R peptides with improved efficacy or biased agonism to reduce side effects, or combination therapies targeting GLP-1 and other gut hormones. Peptides with modification of the N-terminus of exendin-4 produce agonists with distinct effects on receptor trafficking (Jones *et al.* 2018). Agonists that reduced β-arrestin recruitment, receptor internalisation and increased receptor recycling improved insulin release in human islets and glucose control in mice. However, these agonists had no effect on food intake (Jones *et al.* 2018). Another modified agonist (GL0034 based on semaglutide) which had enhanced cAMP potency had similar effects on GLP-1R mediated insulin secretion and weight loss to semaglutide (Jones *et al.* 2022). This indicates that there may be distinct signalling and trafficking pathways involved in GLP-1R-mediated glycaemic control vs appetite suppression. Subsequent studies showed that activated GLP-1R is recruited to membrane nanodomains in a mechanism that involves palmitoylation, and this clustering drives GLP-1R signalling responses and internalisation (Buenaventura *et al.* 2019). Further studies of agonists that selectively modify these distinct signalling pathways could improve GLP-1R targeting therapies for diabetes and obesity. A recent study has proposed that inhibition of the K_ATP_ channels by mutation or long-term use of sulfonylureas leads to persistently depolarised β-cells in diabetes and that GLP-1R switches coupling from Gs-to-Gq signalling to amplify insulin secretion (Oduori *et al.* 2020). Whether GLP-1R agonists can be modified to preferentially activate the Gq pathway and what the physiological consequences are remain to be explored.

The mechanisms by which GLP-1 reduces food intake and promotes weight loss are incompletely understood and probably involve several mechanisms. Some vagal afferents express GLP1R and it was proposed that the effects of GLP-1R agonism on food intake could contribute to the vagal detection of intestinal nutrients. However, animal studies have shown that GLP-1R-expressing neurons in the stomach and the intestines are primarily mechanosensitive, which likely respond to GI distention rather than intestinal GLP-1 activating GLP-1R (Williams *et al.* 2016). As synthetic GLP-1R agonists can penetrate the blood–brain barrier, it is likely that their effects on food intake are driven by direct effects on GLP-1R in the brain. GLP-1R is expressed throughout the CNS and several pathways involving GLP-1R-positive neurons in the hindbrain and hypothalamus have been described to regulate food intake. Studies in mice showed that stimulation of GLP-1-producing NTS neurons that project to the PVN is sufficient to mediate food intake and maintain body weight. Using chemogenetics and slice physiology, GLP-1R in the PVN was shown to augment corticotropin-releasing hormone neuronal excitatory synaptic strength via a mechanism involving PKA-driven enhancement of AMPA receptor membrane trafficking (Liu *et al.* 2017*a*). NTS neurons expressing pre-proglucagon (PPG) also project to the bed nucleus of the stria terminalis, an area associated with feeding, stress and reward behaviour (Williams *et al.* 2018).

A study using fluorescently labelled liraglutide in mice revealed the drug is targeted to POMC neurons and that it indirectly inhibits NPY/AgRP via a GABA-dependent signalling mechanism (Secher *et al.* 2014). A subsequent study that used neuron-specific transgenic mice showed that liraglutide directly activated ARC POMC neurons via TRPC5 calcium channels. GLP-1R was also shown to activate a signalling pathway involving TRPC5 and K_ATP_ channels to enhance the activity of GABAergic neurons, which inhibit NPY/AgRP neurons via post-synaptic GABA-A receptors (He *et al.* 2019). Combined activation of POMC neurons with liraglutide and leptin led to enhanced activation of the TRPC5 pathway indicating that therapies combining agonism of the two receptors may be beneficial in suppressing food intake (He *et al.* 2019). This co-activation probably involves independent activation rather than co-stimulation of signalling pathways as subsequent mouse models showed that leptin receptor and GLP-1R POMC neurons are largely non-overlapping (Biglari *et al.* 2021).

### GIP receptor

Like GLP-1R, GIPR is highly expressed in pancreatic β-cells, increases cAMP-PKA signalling to enhance insulin secretion and suppresses apoptosis which may improve β-cell health (Widenmaier *et al.* 2010). GIP has a bifunctional role in regulating blood glucose. In healthy subjects, GIP has no effect on glucagon responses during hyperglycaemia, whereas GIP increases glucagon and has no effect on insulin secretion during fasting and hypoglycaemia (Tan *et al.* 2013). GIPR also regulates adipocyte function, although findings are contradictory. Mice with deletion of the GIPR are resistant to diet-induced obesity, although chronically elevating GIP has been shown to reduce DIO. Moreover, GIP has been shown to have no effect on weight loss or on energy expenditure in overweight and obese subjects (Finan *et al.* 2013). Differences in the efficacy and pharmacokinetics of agonists and antagonists used in the studies, species differences or GIPR desensitisation could explain some of these discrepancies. In favour of the latter argument, lowering circulating glucose, which is known to promote GIPR desensitisation, improves the insulin response of type-2 diabetic patients treated with GIP and GLP-1, indicating that co-agonism may enhance GLP-1 agonist therapies.

GIPR is expressed at multiple sites within the brain including the ARC, PVN, dorsomedial hypothalamic nucleus (DMH), NTS and area postrema (Adriaenssens *et al.* 2019, Borner *et al.* 2021). Activation of hypothalamic neurons in mice using designer receptors exclusively activated by designer drugs (DREADDs) suppressed food intake (Adriaenssens *et al.* 2019). Co-activation of neurons that co-express *Gipr* and *Glp1r* did not have any further effect on acute food intake than activating *Glp1r* neurons alone (Adriaenssens *et al.* 2019). Subsequent studies have shown that CNS-specific knockout of *GIPR* lowers body weight and improves glucose metabolism and that administration of fatty acyl-GIP induced acute neural activation in key feeding centres of the hypothalamus including ARC, DMH, ventromedial hypothalamus and the lateral hypothalamus providing further evidence that GIP contributes to the central control of energy homeostasis (Zhang *et al.* 2021). Additionally, studies in rodents and shrews have indicated that GIPR in the hindbrain may have an anti-emetic role, although it is unknown whether these neurons contribute to other metabolic functions (Borner *et al.* 2021).

### 5-hydroxytryptamine receptor 2C

Serotonin (5-hydroxytryptamine, 5-HT) within the brain has been known to regulate appetite and body weight for more than 40 years. Compounds that stimulate 5-HT, including d-fenfluramine and sibutramine, were amongst the earliest treatments for obesity; however, they were withdrawn from clinical use due to their side effects including increased heart rate and blood pressure (D'Agostino *et al.* 2018). Mouse knockout studies demonstrated that the GPCR, 5-hydroxytryptamine receptor 2C (5-HT_2C_R), is responsible for the 5-HT effects on feeding, which led to the development of the selective 5-HT_2C_R agonist lorcaserin. Although lorcaserin was clinically approved to improve weight loss, it was withdrawn in 2020 due to a possible increased occurrence of cancer. An improved understanding of how lorcaserin produces its therapeutic effect, and how 5-HT_2C_R controls food intake, could improve future drug design targeting this receptor and improve off-target effects (Wagner *et al.* 2022).

Studies using DREADDs, conditional knockout and reporter mice revealed 5-HT_2C_Rs at the NTS and the ARC are required for 5-HT effects on appetite and food intake and that lorcaserin induces its appetite-suppressive effects by acting upon POMC neurons within these regions (D'Agostino *et al.* 2018). Recently, lorcaserin was shown to also activate PPG NTS neuron populations to reduce food intake (Wagner *et al.* 2022). Using specific agonists of MC4R and GLP-1R, the PPG-NTS and POMC-NTS/ARC neuronal pathways were shown to be functionally independent targets of lorcaserin, indicating that targeting GLP-1R and 5-HT_2C_R may be beneficial. Indeed, combining lorcaserin with liraglutide or exendin-4 produced greater reductions in food intake in mice (Wagner *et al.* 2022), suggesting drugs targeting these two receptors may produce greater effects in obesity. Moreover, as the GLP-1R and MC4R neuronal pathways were shown to be independent, some patients with MC4R mutations may benefit more from GLP-1R agonists than setmelanotide or lorcaserin (Wagner *et al.* 2022).

### Combinatorial GPCR-targeted therapeutics

Observation of improved responses to GLP-1 in combination with other GPCR agonists in animal models led to the development of several co-agonists. As GLP-1 and glucagon have high sequence homology, researchers have designed unimolecular receptor co-agonists that should bind to both receptors. Investigators have designed several such compounds with different ratios of GLP-1 to glucagon. One compound that targets GLP-1R and GCGR, NN-1177, was terminated after the phase 1 clinical trial due to adverse effects including increased pulse rates (Coskun *et al.* 2022). Similar findings were observed for other compounds targeting both receptors (e.g. cotadutide and mazdutide), although when administered in multiple ascending doses, elevations of pulse rate decreased over time (Coskun *et al.* 2022, Ji *et al.* 2022).

Early studies showed that co-agonism using GIP and GLP-1 improved glucose-stimulated insulin release and adiposity in rodents and substantially reduced GI side effects in humans (Finan *et al.* 2013). Tirzepatide, a unimolecular GIP and GLP-1 agonist with greater potency for GIPR than GLP-1R, had enhanced effects on glycaemic control and weight loss than a GLP-1R agonist in type-2 diabetic patients (Khoo & Tan 2020). Once-weekly tirzepatide was also recently shown to improve weight loss in obese patients. However, adverse GI effects were reported in diabetic and obese patients indicating that favouring GIP agonism does not remove these adverse effects (Jastreboff *et al.* 2022).

In parallel with studies investigating GIP and GLP-1 dual agonists, other researchers have developed drugs that inhibit GIPR activity, while activating GLP-1R. AMG-133 is a GIPR-inhibiting antibody conjugated to a GLP-1R agonist, which acts by promoting receptor internalisation to enhance sustained endosomal cAMP responses, resulting in reduced body weight in obese animals (Lu *et al.* 2021). AMG-133 has undergone phase 1 clinical trials, and Amgen press releases have reported up to a 14.5% reduction in body weight in individuals with obesity without diabetes. Similar to other studies, AMG-133 was associated with adverse GI effects; however, the once-monthly administration of AMG-133 may prove advantageous when compared to tirzepatide that requires weekly doses.

A tri-agonist unimolecular peptide, LY3437943, with balanced GLP-1R and GCGR activity, but more GIPR activity, decreased body weight and improved glycaemic control in obese mice. In a first in-human study, LY3437943 achieved similar weight loss effects to tirzepatide and had a substantial effect on appetite control, as well as reductions in fasting triglycerides. Adverse effects including pulse rate were not significantly different to the placebo group (Coskun *et al.* 2022).

Several studies have investigated the utility of combined therapies targeting amylin receptor with other GPCRs, including with GLP-1R. In phase 1 clinical trial, individuals with a BMI > 27 kg/m^2^ were administered once-weekly cagrilinitide in combination with semaglutide subcutaneously. When compared to semaglutide alone, patients receiving both cagrilinitide and semaglutide had greater weight loss, improved glycaemic control and no differences in adverse effects observed (Enebo *et al.* 2021). Further studies will be required in larger cohorts, but this early study indicates that combining drugs targeting amylin receptors and incretin receptors could be beneficial in patients with diabetes and obesity. This is an area of research that is likely to continue to develop as Novo-Nordisk recently announced that its amylin receptor and GLP-1R dual-agonist, Amycretin, is set to enter phase 1 clinical trials in individuals living with obesity. Co-administration of amylin and an MC4R agonist to rats had a greater effect on weight loss and food intake than treatment with amylin alone (Roth *et al.* 2012), and amylin-induced stimulation of thermogenesis in iBAT was blocked by an MC4R antagonist (Li *et al.* 2019), indicating that combined treatment may be beneficial in obesity.

As stated, amylin and CGRP have some cross-affinity at their respective receptors, complicating the deciphering of their respective roles in energy homeostasis. However, their shared effects on glycaemic control and adiposity led to the development of dual amylin-calcitonin receptor agonists (DACRA) that target both receptors with superior potency. The first generation of DACRAs had modest effects on food intake and body weight and improved glycaemic regulation but required frequent (daily) dosing. Recently a long-lasting DACRA, KBP-066A, that can be administered once weekly was described. In rats, KBP-066A improved fasting blood glucose, plasma insulin and oral glucose tolerance and reduced body weight in obese diabetic rats (Andreassen *et al.* 2021), indicating that a regime that is more likely to be tolerated by patients still has a beneficial effect on energy homeostasis. Encouragingly, combined treatment of high-fat diet-fed rats with a short-acting DACRA (KBP-089) and the GLP1R agonist liraglutide had an additive effect, lowering body weight, reducing fat depot sizes, improving glucose metabolism and gastric emptying (Larsen *et al.* 2021).

## Conclusions

An increased understanding of how appetite is controlled, and the GPCRs that regulate these physiological pathways, has led to a promising number of targets for the treatment of diabetes and obesity. Drugs that target several of these GPCRs have already been approved, while many more are in development or have been tested in pre-clinical models and clinical trials. However, the deletion of GPCR genes in animal models and agonism/antagonism of individual receptors have demonstrated that many hormonal pathways converge to regulate appetite and the complexities of these pathways remain incompletely understood. Targeting multiple GPCRs simultaneously may improve treatments, and early reports of combination drugs targeting GCGR/GLP-1R/GIPR show promising additive improvements in weight loss. Additionally, the unimolecular nature of many of these compounds, rather than multiple individual GPCR-targeting drugs may improve patient compliance. It is likely many more combinatorial drug strategies will emerge in the coming years.

Despite promising developments, many GPCR-targeting drugs for the treatment of diabetes and obesity are associated with side effects and these may reduce patient compliance. More research is required to understand how different GPCR signalling pathways regulate functions in individual tissues. An increased understanding of these nuanced differences in signalling could inform the design of biased ligands that reduce or remove off-target effects. Additionally, large-scale genetics projects investigating rare coding variants associated with obesity have yielded new GPCR targets, while GPCRs in peripheral tissues involved in energy homeostasis (e.g. free fatty acid receptors in adipose tissue) could emerge as new targets for the next generation of drugs for metabolic disease. There is still clearly much work to do in this area.

## Declaration of interest

The authors have no conflicts of interest to declare.

## Funding

This work was supported by a Sir Henry Dale Fellowship jointly funded by the Wellcome Trusthttp://dx.doi.org/10.13039/100010269 and the Royal Societyhttp://dx.doi.org/10.13039/501100000288 (Grant Number 224155/Z/21/Z to CMG).

## References

[bib1] Adriaenssens AE Biggs EK Darwish T Tadross J Sukthankar T Girish M Polex-Wolf J Lam BY Zvetkova I Pan W, 2019 Glucose-dependent insulinotropic polypeptide receptor-expressing cells in the hypothalamus regulate food intake. Cell Metabolism 30 987–996.e6. (10.1016/j.cmet.2019.07.013) PMC6838660 31447324

[bib2] Andreassen KV Larsen AT Sonne N Mohamed KE Karsdal MA & Henriksen K 2021 KBP-066A, a long-acting dual amylin and calcitonin receptor agonist, induces weight loss and improves glycemic control in obese and diabetic rats. Molecular Metabolism 53 101282. (10.1016/j.molmet.2021.101282) PMC8313742 34214708

[bib3] Aronne LJ Halseth AE Burns CM Miller S & Shen LZ 2010 Enhanced weight loss following coadministration of pramlintide with sibutramine or phentermine in a multicenter trial. Obesity 18 1739–1746. (10.1038/oby.2009.478) 20094043

[bib4] Baron M Maillet J Huyvaert M Dechaume A Boutry R Loiselle H Durand E Toussaint B Vaillant E Philippe J, 2019 Loss-of-function mutations in MRAP2 are pathogenic in hyperphagic obesity with hyperglycemia and hypertension. Nature Medicine 25 1733–1738. (10.1038/s41591-019-0622-0) PMC6858878 31700171

[bib5] Beaudry JL Kaur KD Varin EM Baggio LL Cao X Mulvihill EE Stern JH Campbell JE Scherer PE & Drucker DJ 2019 The brown adipose tissue glucagon receptor is functional but not essential for control of energy homeostasis in mice. Molecular Metabolism 22 37–48. (10.1016/j.molmet.2019.01.011) 30772257 PMC6437632

[bib6] Biglari N Gaziano I Schumacher J Radermacher J Paeger L Klemm P Chen W Corneliussen S Wunderlich CM Sue M, 2021 Functionally distinct POMC-expressing neuron subpopulations in hypothalamus revealed by intersectional targeting. Nature Neuroscience 24 913–929. (10.1038/s41593-021-00854-0) 34002087 PMC8249241

[bib7] Borner T Geisler CE Fortin SM Cosgrove R Alsina-Fernandez J Dogra M Doebley S Sanchez-Navarro MJ Leon RM Gaisinsky J, 2021 GIP receptor agonism attenuates GLP-1 receptor agonist-induced nausea and emesis in preclinical models. Diabetes 70 2545–2553. (10.2337/db21-0459) 34380697 PMC8564411

[bib8] Brouwers B de Oliveira EM Marti-Solano M Monteiro FBF Laurin SA Keogh JM Henning E Bounds R Daly CA Houston S, 2021 Human MC4R variants affect endocytosis, trafficking and dimerization revealing multiple cellular mechanisms involved in weight regulation. Cell Reports 34 108862. (10.1016/j.celrep.2021.108862) PMC7994375 33761344

[bib9] Buenaventura T Bitsi S Laughlin WE Burgoyne T Lyu Z Oqua AI Norman H McGlone ER Klymchenko AS Correa IR Jr, 2019 Agonist-induced membrane nanodomain clustering drives GLP-1 receptor responses in pancreatic beta cells. PLOS Biology 17 e3000097. (10.1371/journal.pbio.3000097) PMC6716783 31430273

[bib10] Campos CA Bowen AJ Schwartz MW & Palmiter RD 2016 Parabrachial CGRP neurons control meal termination. Cell Metabolism 23 811–820. (10.1016/j.cmet.2016.04.006) 27166945 PMC4867080

[bib11] Coester B Pence SW Arrigoni S Boyle CN Le Foll C & Lutz TA 2020 RAMP1 and RAMP3 differentially control Amylin’s effects on food intake, glucose and energy balance in male and female mice. Neuroscience 447 74–93. (10.1016/j.neuroscience.2019.11.036) 31881259

[bib12] Collet TH Dubern B Mokrosinski J Connors H Keogh JM Mendes de Oliveira E Henning E Poitou-Bernert C Oppert JM Tounian P, 2017 Evaluation of a melanocortin-4 receptor (MC4R) agonist (Setmelanotide) in MC4R deficiency. Molecular Metabolism 6 1321–1329. (10.1016/j.molmet.2017.06.015) 29031731 PMC5641599

[bib13] Coskun T Urva S Roell WC Qu H Loghin C Moyers JS O'Farrell LS Briere DA Sloop KW Thomas MK, 2022 LY3437943, a novel triple glucagon, GIP, and GLP-1 receptor agonist for glycemic control and weight loss: from discovery to clinical proof of concept. Cell Metabolism 34 1234–1247.e9. (10.1016/j.cmet.2022.07.013) 35985340

[bib14] D'Agostino G Lyons D Cristiano C Lettieri M Olarte-Sanchez C Burke LK Greenwald-Yarnell M Cansell C Doslikova B Georgescu T, 2018 Nucleus of the solitary tract serotonin 5-HT(2C) receptors modulate food intake. Cell Metabolism 28 619–630.e5. (10.1016/j.cmet.2018.07.017) PMC6371983 30146485

[bib15] Davis EA Wald HS Suarez AN Zubcevic J Liu CM Cortella AM Kamitakahara AK Polson JW Arnold M Grill HJ, 2020 Ghrelin signaling affects feeding behavior, metabolism, and memory through the vagus nerve. Current Biology 30 4510–4518.e6. (10.1016/j.cub.2020.08.069) 32946754 PMC7674191

[bib16] Enebo LB Berthelsen KK Kankam M Lund MT Rubino DM Satylganova A & Lau DCW 2021 Safety, tolerability, pharmacokinetics, and pharmacodynamics of concomitant administration of multiple doses of cagrilintide with semaglutide 2.4 mg for weight management: a randomised, controlled, phase 1b trial. Lancet 397 1736–1748. (10.1016/S0140-6736(2100845-X) 33894838

[bib17] Esler WP Rudolph J Claus TH Tang W Barucci N Brown SE Bullock W Daly M DeCarr L Li Y, 2007 Small-molecule ghrelin receptor antagonists improve glucose tolerance, suppress appetite, and promote weight loss. Endocrinology 148 5175–5185. (10.1210/en.2007-0239) 17656463

[bib18] Finan B Ma T Ottaway N Muller TD Habegger KM Heppner KM Kirchner H Holland J Hembree J Raver C, 2013 Unimolecular dual incretins maximize metabolic benefits in rodents, monkeys, and humans. Science Translational Medicine 5 209ra151. (10.1126/scitranslmed.3007218) 24174327

[bib19] Garnsey MR Smith AC Polivkova J Arons AL Bai G Blakemore C Boehm M Buzon LM Campion SN Cerny M, 2023 Discovery of the potent and selective MC4R antagonist PF-07258669 for the potential treatment of appetite loss. Journal of Medicinal Chemistry 66 3195–3211. (10.1021/acs.jmedchem.2c02012) 36802610

[bib20] Ge X Yang H Bednarek MA Galon-Tilleman H Chen P Chen M Lichtman JS Wang Y Dalmas O Yin Y, 2018 LEAP2 is an endogenous antagonist of the ghrelin receptor. Cell Metabolism 27 461–469.e6. (10.1016/j.cmet.2017.10.016) 29233536

[bib21] Geary N Le Sauter J & Noh U 1993 Glucagon acts in the liver to control spontaneous meal size in rats. American Journal of Physiology 264 R116–R122. (10.1152/ajpregu.1993.264.1.R116) 8430871

[bib22] Gedulin BR Jodka CM Herrmann K & Young AA 2006 Role of endogenous amylin in glucagon secretion and gastric emptying in rats demonstrated with the selective antagonist, AC187. Regulatory Peptides 137 121–127. (10.1016/j.regpep.2006.06.004) 16914214

[bib23] Ghamari-Langroudi M Cakir I Lippert RN Sweeney P Litt MJ Ellacott KLJ & Cone RD 2018 Regulation of energy rheostasis by the melanocortin-3 receptor. Science Advances 4 eaat0866. (10.1126/sciadv.aat0866) PMC6105298 30140740

[bib24] Granell S Mohammad S Ramanagoudr-Bhojappa R & Baldini G 2010 Obesity-linked variants of melanocortin-4 receptor are misfolded in the endoplasmic reticulum and can be rescued to the cell surface by a chemical chaperone. Molecular Endocrinology 24 1805–1821. (10.1210/me.2010-0071) 20631012 PMC2940480

[bib25] Greenfield JR Miller JW Keogh JM Henning E Satterwhite JH Cameron GS Astruc B Mayer JP Brage S See TC, 2009 Modulation of blood pressure by central melanocortinergic pathways. New England Journal of Medicine 360 44–52. (10.1056/NEJMoa0803085) 19092146

[bib26] Hagemann CA Jensen MS Holm S Gasbjerg LS Byberg S Skov-Jeppesen K Hartmann B Holst JJ Dela F Vilsbøll T, 2022 LEAP2 reduces postprandial glucose excursions and ad libitum food intake in healthy men. Cell Reports. Medicine 3 100582. (10.1016/j.xcrm.2022.100582) PMC9043997 35492241

[bib27] Halloran J Lalande A Zang M Chodavarapu H & Riera CE 2020 Monoclonal therapy against calcitonin gene-related peptide lowers hyperglycemia and adiposity in type 2 diabetes mouse models. Metabolism Open 8 100060. (10.1016/j.metop.2020.100060) PMC7566843 33089134

[bib28] Hay DL Garelja ML Poyner DR & Walker CS 2018 Update on the pharmacology of calcitonin/CGRP family of peptides: IUPHAR Review 25. British Journal of Pharmacology 175 3–17. (10.1111/bph.14075) 29059473 PMC5740251

[bib29] He Z Gao Y Lieu L Afrin S Cao J Michael NJ Dong Y Sun J Guo H & Williams KW 2019 Direct and indirect effects of liraglutide on hypothalamic POMC and NPY/AgRP neurons - Implications for energy balance and glucose control. Molecular Metabolism 28 120–134. (10.1016/j.molmet.2019.07.008) 31446151 PMC6822260

[bib30] Heyder NA Kleinau G Speck D Schmidt A Paisdzior S Szczepek M Bauer B Koch A Gallandi M Kwiatkowski D, 2021 Structures of active melanocortin-4 receptor-Gs-protein complexes with NDP-alpha-MSH and setmelanotide. Cell Research 31 1176–1189. (10.1038/s41422-021-00569-8) 34561620 PMC8563958

[bib31] Janah L Kjeldsen S Galsgaard KD Winther-Sørensen M Stojanovska E Pedersen J Knop FK Holst JJ & Wewer Albrechtsen NJ 2019 Glucagon receptor signaling and glucagon resistance. International Journal of Molecular Sciences 20 3314. (10.3390/ijms20133314) PMC6651628 31284506

[bib32] Jastreboff AM Aronne LJ Ahmad NN Wharton S Connery L Alves B Kiyosue A Zhang S Liu B Bunck MC, 2022 Tirzepatide once weekly for the treatment of obesity. New England Journal of Medicine 387 205–216. (10.1056/NEJMoa2206038) 35658024

[bib33] Ji L Gao L Jiang H Yang J Yu L Wen J Cai C Deng H Feng L Song B, 2022 Safety and efficacy of a GLP-1 and glucagon receptor dual agonist mazdutide (IBI362) 9 mg and 10 mg in Chinese adults with overweight or obesity: a randomised, placebo-controlled, multiple-ascending-dose phase 1b trial. EClinicalmedicine 54 101691. (10.1016/j.eclinm.2022.101691) PMC9561728 36247927

[bib34] Jones B Buenaventura T Kanda N Chabosseau P Owen BM Scott R Goldin R Angkathunyakul N Corrêa IR Jr Bosco D, 2018 Targeting GLP-1 receptor trafficking to improve agonist efficacy. Nature Communications 9 1602. (10.1038/s41467-018-03941-2) PMC5913239 29686402

[bib35] Jones B Burade V Akalestou E Manchanda Y Ramchunder Z Carrat G Nguyen-Tu MS Marchetti P Piemonti L Leclerc I, 2022 In vivo and in vitro characterization of GL0034, a novel long-acting glucagon-like peptide-1 receptor agonist. Diabetes, Obesity and Metabolism 24 2090–2101. (10.1111/dom.14794) PMC9796023 35676825

[bib36] Kalra SP Ueno N & Kalra PS 2005 Stimulation of appetite by ghrelin is regulated by leptin restraint: peripheral and central sites of action. Journal of Nutrition 135 1331–1335. (10.1093/jn/135.5.1331) 15867335

[bib37] Khoo B & Tan TM 2020 Combination gut hormones: prospects and questions for the future of obesity and diabetes therapy. Journal of Endocrinology 246 R65–R74. (10.1530/JOE-20-0119) 32438346

[bib38] Kievit P Halem H Marks DL Dong JZ Glavas MM Sinnayah P Pranger L Cowley MA Grove KL & Culler MD 2013 Chronic treatment with a melanocortin-4 receptor agonist causes weight loss, reduces insulin resistance, and improves cardiovascular function in diet-induced obese rhesus macaques. Diabetes 62 490–497. (10.2337/db12-0598) 23048186 PMC3554387

[bib39] Kim SJ Nian C Karunakaran S Clee SM Isales CM & McIntosh CH 2012 GIP-overexpressing mice demonstrate reduced diet-induced obesity and steatosis, and improved glucose homeostasis. PLoS One 7 e40156. (10.1371/journal.pone.0040156) PMC3388996 22802954

[bib40] Lam BYH Williamson A Finer S Day FR Tadross JA Gonçalves Soares A Wade K Sweeney P Bedenbaugh MN Porter DT, 2021 MC3R links nutritional state to childhood growth and the timing of puberty. Nature 599 436–441. (10.1038/s41586-021-04088-9) 34732894 PMC8819628

[bib41] Larsen AT Gydesen S Sonne N Karsdal MA & Henriksen K 2021 The dual amylin and calcitonin receptor agonist KBP-089 and the GLP-1 receptor agonist liraglutide act complimentarily on body weight reduction and metabolic profile. BMC Endocrine Disorders 21 10. (10.1186/s12902-020-00678-2) 33413317 PMC7791885

[bib42] Lau DCW Erichsen L Francisco AM Satylganova A le Roux CW McGowan B Pedersen SD Pietilainen KH Rubino D & Batterham RL 2021 Once-weekly cagrilintide for weight management in people with overweight and obesity: a multicentre, randomised, double-blind, placebo-controlled and active-controlled, dose-finding phase 2 trial. Lancet 398 2160–2172. (10.1016/S0140-6736(2101751-7) 34798060

[bib43] Li X Fan K Li Q Pan D Hai R & Du C 2019 Melanocortin 4 receptor–mediated effects of amylin on thermogenesis and regulation of food intake. Diabetes/Metabolism Research and Reviews 35 e3149. (10.1002/dmrr.3149) 30851142

[bib44] Li YQ Shrestha Y Pandey M Chen M Kablan A Gavrilova O Offermanns S & Weinstein LS 2016 G(q/11)α and G(s)α mediate distinct physiological responses to central melanocortins. Journal of Clinical Investigation 126 40–49. (10.1172/JCI76348) 26595811 PMC4701544

[bib45] Liu J Conde K Zhang P Lilascharoen V Xu Z Lim BK Seeley RJ Zhu JJ Scott MM & Pang ZP 2017a Enhanced AMPA receptor trafficking mediates the anorexigenic effect of endogenous glucagon-like Peptide-1 in the paraventricular hypothalamus. Neuron 96 897–909.e5. (10.1016/j.neuron.2017.09.042) PMC5729931 29056294

[bib46] Liu T Kamiyoshi A Sakurai T Ichikawa-Shindo Y Kawate H Yang L Tanaka M Xian X Imai A Zhai L, 2017b Endogenous calcitonin gene-related peptide regulates lipid metabolism and energy homeostasis in male mice. Endocrinology 158 1194–1206. (10.1210/en.2016-1510) 28324021

[bib47] Lotta LA Mokrosiński J Mendes de Oliveira E Li C Sharp SJ Luan J Brouwers B Ayinampudi V Bowker N Kerrison N, 2019 Human gain-of-function MC4R variants show signaling bias and protect against obesity. Cell 177 597–607.e9. (10.1016/j.cell.2019.03.044) 31002796 PMC6476272

[bib48] Lu SC Chen M Atangan L Killion EA Komorowski R Cheng Y Netirojjanakul C Falsey JR Stolina M Dwyer D, 2021 GIPR antagonist antibodies conjugated to GLP-1 peptide are bispecific molecules that decrease weight in obese mice and monkeys. Cell Reports. Medicine 2 100263. (10.1016/j.xcrm.2021.100263) PMC8149376 34095876

[bib49] Makwana K Chodavarapu H Morones N Chi J Barr W Novinbakht E Wang Y Nguyen PT Jovanovic P Cohen P, 2021 Sensory neurons expressing calcitonin gene-related peptide α regulate adaptive thermogenesis and diet-induced obesity. Molecular Metabolism 45 101161. (10.1016/j.molmet.2021.101161) PMC7820934 33412345

[bib50] Mani BK Puzziferri N He Z Rodriguez JA Osborne-Lawrence S Metzger NP Chhina N Gaylinn B Thorner MO Thomas EL, 2019 LEAP2 changes with body mass and food intake in humans and mice. Journal of Clinical Investigation 129 3909–3923. (10.1172/JCI125332) 31424424 PMC6715358

[bib51] McLean BA Wong CK Campbell JE Hodson DJ Trapp S & Drucker DJ 2021 Revisiting the complexity of GLP-1 action from sites of synthesis to receptor activation. Endocrine Reviews 42 101–132. (10.1210/endrev/bnaa032) 33320179 PMC7958144

[bib52] Mighiu PI Yue JT Filippi BM Abraham MA Chari M Lam CK Yang CS Christian NR Charron MJ & Lam TK 2013 Hypothalamic glucagon signaling inhibits hepatic glucose production. Nature Medicine 19 766–772. (10.1038/nm.3115) 23685839

[bib53] Müller TD Nogueiras R Andermann ML Andrews ZB Anker SD Argente J Batterham RL Benoit SC Bowers CY Broglio F, 2015 Ghrelin. Molecular Metabolism 4 437–460. (10.1016/j.molmet.2015.03.005) 26042199 PMC4443295

[bib54] Nason SR Antipenko J Presedo N Cunningham SE Pierre TH Kim T Paul JR Holleman C Young ME Gamble KL, 2021 Glucagon receptor signaling regulates weight loss via central KLB receptor complexes. JCI Insight 6 e141323. (10.1172/jci.insight.141323) 33411693 PMC7934938

[bib55] Nilsson C Hansen TK Rosenquist C Hartmann B Kodra JT Lau JF Clausen TR Raun K & Sams A 2016 Long acting analogue of the calcitonin gene-related peptide induces positive metabolic effects and secretion of the glucagon-like peptide-1. European Journal of Pharmacology 773 24–31. (10.1016/j.ejphar.2016.01.003) 26808305

[bib56] Oduori OS Murao N Shimomura K Takahashi H Zhang Q Dou H Sakai S Minami K Chanclon B Guida C, 2020 Gs/Gq signaling switch in beta cells defines incretin effectiveness in diabetes. Journal of Clinical Investigation 130 6639–6655. (10.1172/JCI140046) 33196462 PMC7685756

[bib57] Ouhilal S Vuguin P Cui L Du XQ Gelling RW Reznik SE Russell R Parlow AF Karpovsky C Santoro N, 2012 Hypoglycemia, hyperglucagonemia, and fetoplacental defects in glucagon receptor knockout mice: a role for glucagon action in pregnancy maintenance. American Journal of Physiology. Endocrinology and Metabolism 302 E522–E531. (10.1152/ajpendo.00420.2011) 22167521 PMC3311287

[bib58] Portha B Tourrel-Cuzin C & Movassat J 2011 Activation of the GLP-1 receptor signalling pathway: a relevant strategy to repair a deficient beta-cell mass. Experimental Diabetes Research 2011 376509. (10.1155/2011/376509) 21716694 PMC3118608

[bib59] Quiñones M Al-Massadi O Gallego R Fernø J Diéguez C López M & Nogueiras R 2015 Hypothalamic CaMKKβ mediates glucagon anorectic effect and its diet-induced resistance. Molecular Metabolism 4 961–970. (10.1016/j.molmet.2015.09.014) 26909312 PMC4731730

[bib60] Roth JD D'Souza L Griffin PS Athanacio J Trevaskis JL Nazarbaghi R Jodka C Athanacio J Hoyt J Forood B, 2012 Interactions of amylinergic and melanocortinergic systems in the control of food intake and body weight in rodents. Diabetes, Obesity and Metabolism 14 608–615. (10.1111/j.1463-1326.2012.01570.x) 22276636

[bib61] Rouault AA Srinivasan DK Yin TC Lee AA & Sebag JA 2017 Melanocortin receptor accessory proteins (MRAPs): functions in the melanocortin system and beyond. Biochimica et Biophysica Acta (BBA)-Molecular Basis of Disease 1863 2462–2467. (10.1016/j.bbadis.2017.05.008) 28499989

[bib62] Sanford D Luong L Gabalski A Oh S Vu JP Pisegna JR & Germano P 2019 An intraperitoneal treatment with calcitonin gene-related peptide (CGRP) regulates appetite, energy intake/expenditure, and metabolism. Journal of Molecular Neuroscience 67 28–37. (10.1007/s12031-018-1202-3) 30535790 PMC6736536

[bib63] Secher A Jelsing J Baquero AF Hecksher-Sørensen J Cowley MA Dalbøge LS Hansen G Grove KL Pyke C Raun K, 2014 The arcuate nucleus mediates GLP-1 receptor agonist liraglutide-dependent weight loss. Journal of Clinical Investigation 124 4473–4488. (10.1172/JCI75276) 25202980 PMC4215190

[bib64] Spana C Jordan R & Fischkoff S 2022 Effect of Bremelanotide on body weight of obese women: data from two phase 1 randomized controlled trials. Diabetes, Obesity and Metabolism 24 1084–1093. (10.1111/dom.14672) PMC9314948 35170192

[bib65] Srisai D Yin TC Lee AA Rouault AAJ Pearson NA Grobe JL & Sebag JA 2017 MRAP2 regulates ghrelin receptor signaling and hunger sensing. Nature Communications 8 713. (10.1038/s41467-017-00747-6) PMC5620068 28959025

[bib66] Sutton GM Perez-Tilve D Nogueiras R Fang J Kim JK Cone RD Gimble JM Tschop MH & Butler AA 2008 The melanocortin-3 receptor is required for entrainment to meal intake. Journal of Neuroscience 28 12946–12955. (10.1523/JNEUROSCI.3615-08.2008) 19036988 PMC2613653

[bib67] Sweeney P Bedenbaugh MN Maldonado J Pan P Fowler K Williams SY Gimenez LE Ghamari-Langroudi M Downing G Gui Y, 2021 The melanocortin-3 receptor is a pharmacological target for the regulation of anorexia. Science Translational Medicine 13 eabd6434. (10.1126/scitranslmed.abd6434) 33883274 PMC9022017

[bib68] Tan TM Field BC McCullough KA Troke RC Chambers ES Salem V Gonzalez Maffe J Baynes KC De Silva A Viardot A, 2013 Coadministration of glucagon-like peptide-1 during glucagon infusion in humans results in increased energy expenditure and amelioration of hyperglycemia. Diabetes 62 1131–1138. (10.2337/db12-0797) 23248172 PMC3609580

[bib69] Tian J Guo L Sui S Driskill C Phensy A Wang Q Gauba E Zigman JM Swerdlow RH Kroener S, 2019 Disrupted hippocampal growth hormone secretagogue receptor 1alpha interaction with dopamine receptor D1 plays a role in Alzheimer's disease. Science Translational Medicine 11 eaav6278. (10.1126/scitranslmed.aav6278) 31413143 PMC6776822

[bib70] Townsend LK Medak KD Knuth CM Peppier WT Charron MJ & Wright DC 2019 Loss of glucagon signaling alters white adipose tissue browning. FASEB Journal 33 4824–4835. (10.1096/fj.201802048RR) 30615494

[bib72] Vasileva A Marx T Beaudry JL & Stern JH 2022 Glucagon receptor signaling at white adipose tissue does not regulate lipolysis. American Journal of Physiology. Endocrinology and Metabolism 323 E389–E401. (10.1152/ajpendo.00078.2022) 36002172 PMC9576180

[bib73] Wagner S Brierley DI Leeson-Payne A Jiang W Chianese R Lam BYH Dowsett GKC Cristiano C Lyons D Reimann F, 2022 Obesity medication lorcaserin activates brainstem GLP-1 neurons to reduce food intake and augments GLP-1 receptor agonist induced appetite suppression. Molecular Metabolism 68 101665. (10.1016/j.molmet.2022.101665) PMC9841057 36592795

[bib74] Widenmaier SB Kim SJ Yang GK De Los Reyes T Nian C Asadi A Seino Y Kieffer TJ Kwok YN & McIntosh CH 2010 A GIP receptor agonist exhibits beta-cell anti-apoptotic actions in rat models of diabetes resulting in improved beta-cell function and glycemic control. PLoS One 5 e9590. (10.1371/journal.pone.0009590) PMC2834736 20231880

[bib75] Williams DL Lilly NA Edwards IJ Yao P Richards JE & Trapp S 2018 GLP-1 action in the mouse bed nucleus of the stria terminalis. Neuropharmacology 131 83–95. (10.1016/j.neuropharm.2017.12.007) 29221794 PMC5840513

[bib76] Williams EK Chang RB Strochlic DE Umans BD Lowell BB & Liberles SD 2016 Sensory neurons that detect stretch and nutrients in the digestive system. Cell 166 209–221. (10.1016/j.cell.2016.05.011) 27238020 PMC4930427

[bib77] Yeo GSH Chao DHM Siegert AM Koerperich ZM Ericson MD Simonds SE Larson CM Luquet S Clarke I Sharma S, 2021 The melanocortin pathway and energy homeostasis: from discovery to obesity therapy. Molecular Metabolism 48 101206. (10.1016/j.molmet.2021.101206) PMC8050006 33684608

[bib78] Yin TC Bauchle CJ Rouault AAJ Stephens SB & Sebag JA 2020 The Insulinostatic effect of ghrelin requires MRAP2 expression in delta cells. iScience 23 101216. (10.1016/j.isci.2020.101216) PMC7300157 32535024

[bib79] Zakariassen HL John LM Lykkesfeldt J Raun K Glendorf T Schaffer L Lundh S Secher A Lutz TA & Le Foll C 2020 Salmon calcitonin distributes into the arcuate nucleus to a subset of NPY neurons in mice. Neuropharmacology 167 107987. (10.1016/j.neuropharm.2020.107987) 32035146

[bib80] Zhang Q Delessa CT Augustin R Bakhti M Colldén G Drucker DJ Feuchtinger A Caceres CG Grandl G Harger A, 2021 The glucose-dependent insulinotropic polypeptide (GIP) regulates body weight and food intake via CNS-GIPR signaling. Cell Metabolism 33 833–844.e5. (10.1016/j.cmet.2021.01.015) 33571454 PMC8035082

[bib81] Zhao X Wang M Wen Z Lu Z Cui L Fu C Xue H Liu Y & Zhang Y 2021 GLP-1 receptor agonists: beyond their pancreatic effects. Frontiers in Endocrinology 12 721135. (10.3389/fendo.2021.721135) 34497589 PMC8419463

[bib82] Zhu X Callahan MF Gruber KA Szumowski M & Marks DL 2020 Melanocortin-4 receptor antagonist TCMCB07 ameliorates cancer- and chronic kidney disease-associated cachexia. Journal of Clinical Investigation 130 4921–4934. (10.1172/JCI138392) 32544087 PMC7456235

